# William H. Atkinson, M.D., D.D.S.

**Published:** 1891-05

**Authors:** 


### WILLIAM H. ATKINSON, M.D., D.D.S.

We are only able to announce in this issue of the Journal that Dr. W. H. Atkinson died on April 2. He had had a severe attack of grippe which ended in pneumonia.

Dr. Atkinson was born January 23, 1815, and was, therefore, seventy-six years of age at the time of his death.

The work of his life has thus extended over a series of years the most important in dentistry, and in which all that goes to make it worthy to be called a profession has been accomplished.

We hope to be able to give in our next number a full account of his life-work.

